# Supplementary material for: Association of Midgut Bacteria and Their Metabolic Pathways with Zika Infection and Insecticide Resistance in Colombian Aedes aegypti Populations
Source: Viruses. 2022 Oct 6;14(10):2197. doi: 10.3390/v14102197 (PMC9609292; doi:10.3390/v14102197)
Supplement: Supplementary file 1 [file viruses-14-02197-s001.zip › Supplementary_Material_Paper ZIKV 20_08_2022.pdf]

Supplementary Material

Supplementary Figures

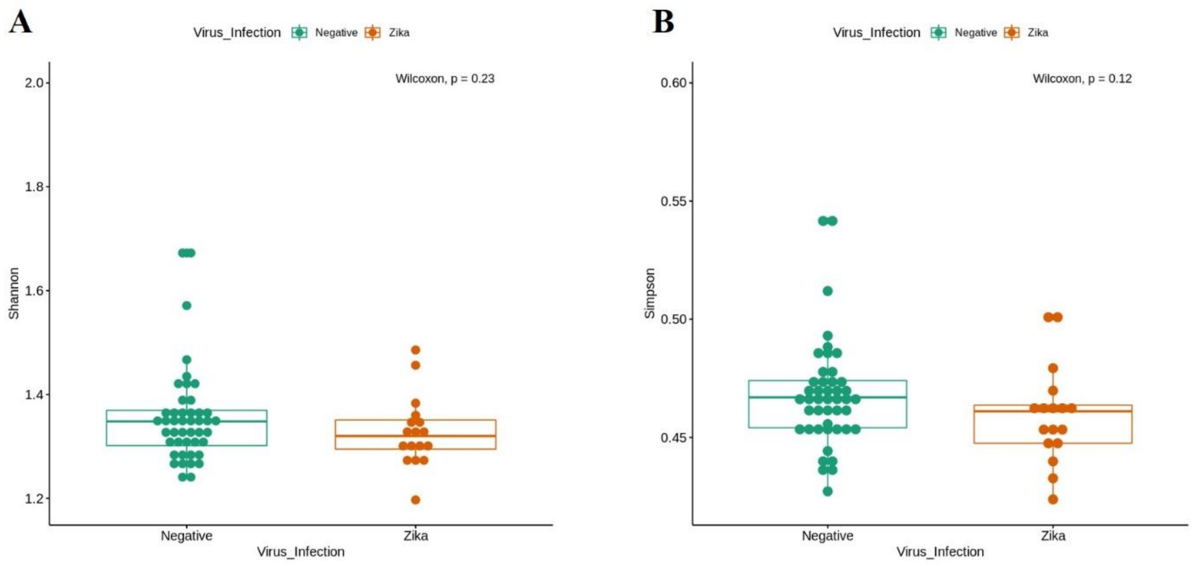

Supplementary Figure S1. Comparison of bacterial diversity in the midgut of ZIKV-infected and non-infected *Ae. aegypti*. Shannon (A) and Simpson (B) alpha diversity indices are shown. Wilcoxon rank-sum tests were conducted under R statistical software version 4.1.2. Groups were not significantly different when $p > 0.05$.

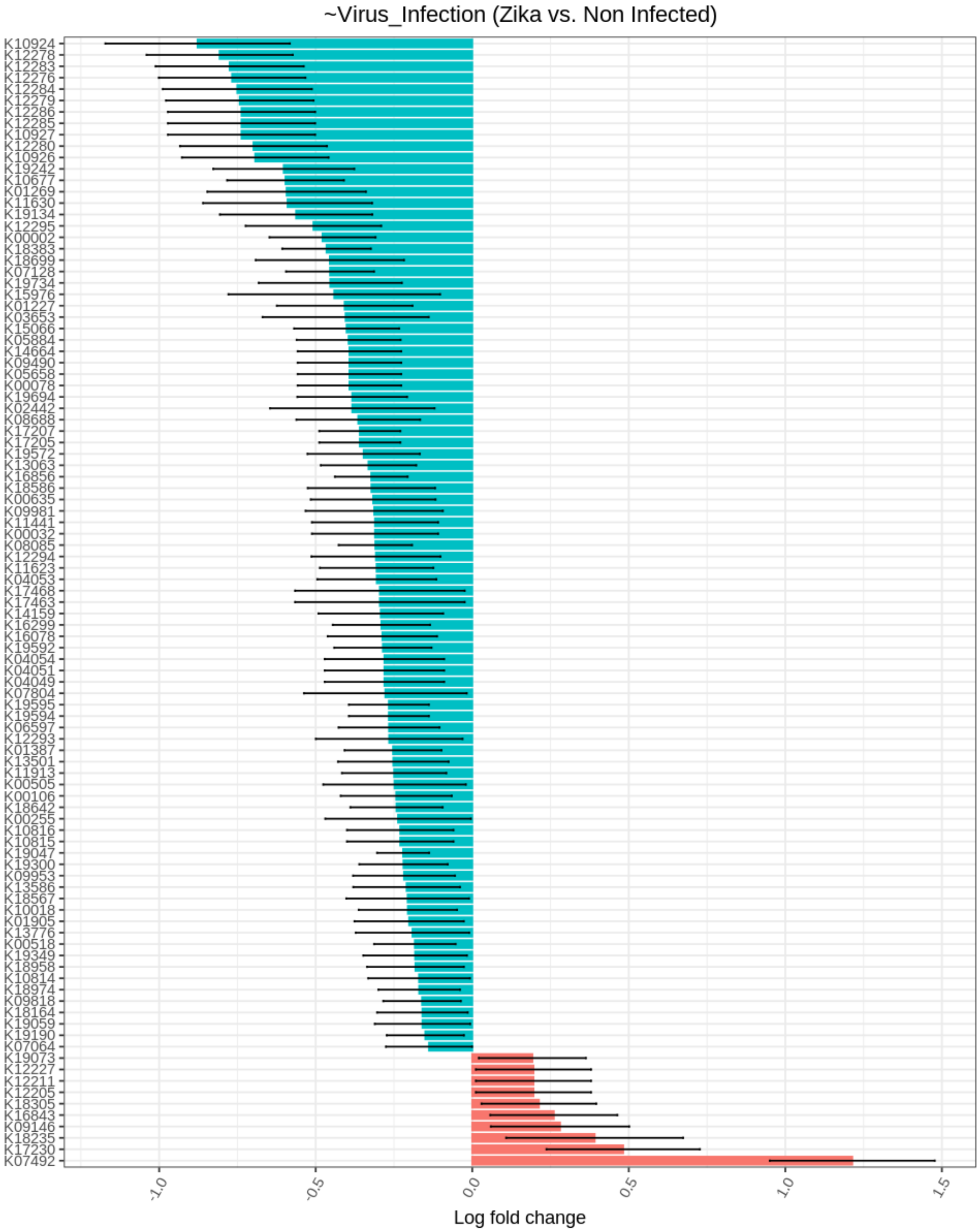

Supplementary Figure S2. KEGG genes with significantly different abundance in *Ae. aegypti* midguts stratified by ZIKV infection using ANCOM-BC ($p < 0.05$).

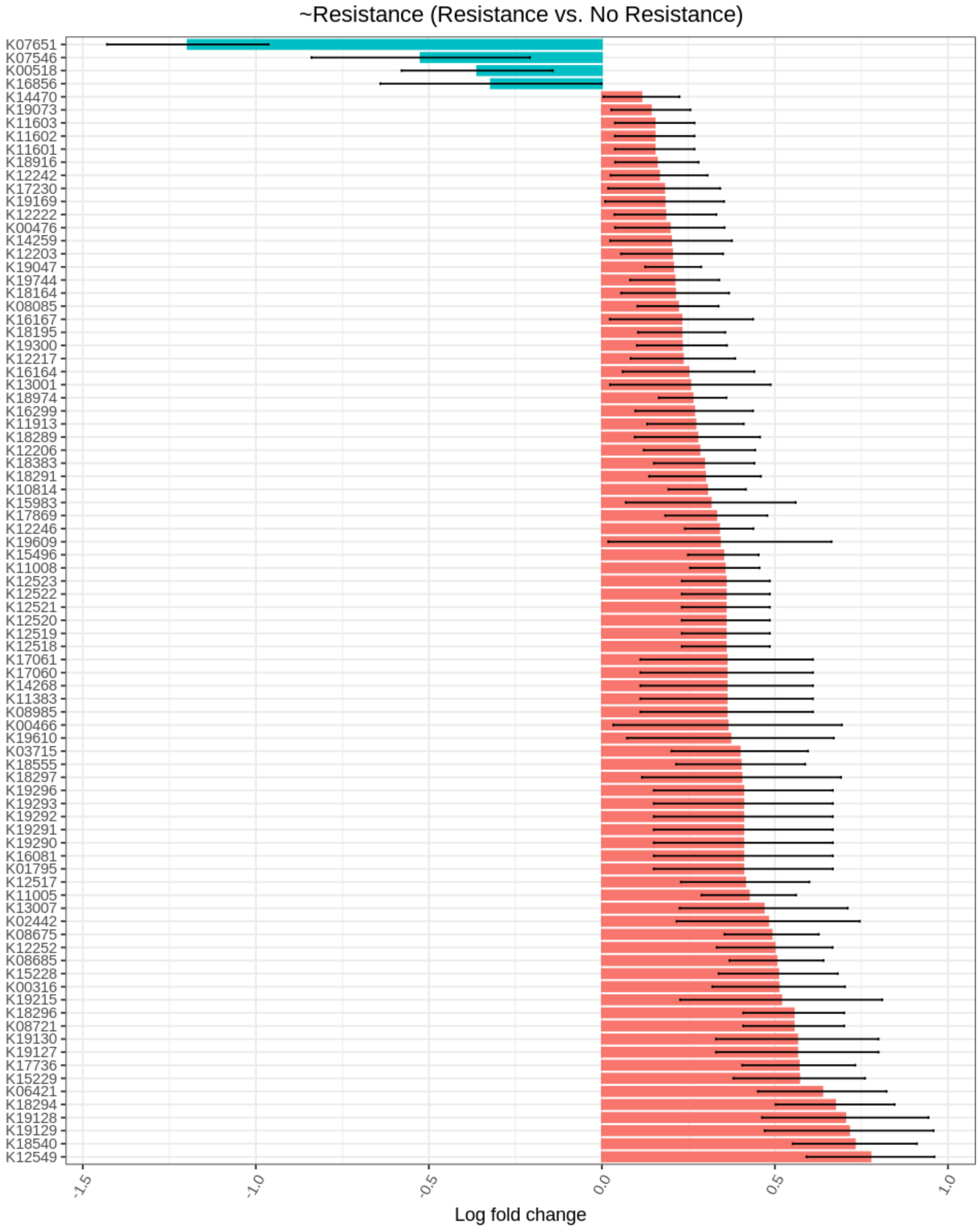

Supplementary Figure S3. KEGG genes with significantly different abundance in *Ae. aegypti* midguts stratified by lambda-cyhalothrin resistance using ANCOM-BC ($p < 0.05$).

Supplementary Tables

Supplementary Table S1. Principal KEGG pathways associated with ZIKV infection in *Ae. aegypti* midguts.

Supplementary Table S2. Principal KEGG pathways associated with lambda-cyhalothrin resistance in *Ae. aegypti* midguts.
